# Interesting Experiments upon the Body of Heidenblut the Murderer

**Published:** 1875-03-15

**Authors:** 

**Affiliations:** Philadelphia


### INTERESTING EXPERIMENTS UPON THE BODY OF HEIDENBLUT, THE MURDERER.

*Philadelphia Correspondence, Boston Med. and Surg. Jour., Feb.* 25, 1875.

INTERESTING experiments were performed a few days since upon the body of Heidenblut, the murderer, directly after his remains were removed from the gallows. Those upon the laryngeal nerves were instituted at the request of Dr. Weir Mitchell, and were skilfully performed by Dr. W.W. Keen, with the assistance of Dr. Carl Seiler. In the *Medical Times and Gazette* for December 19, 1874, was published a paper read by Dr. George Johnson before the Royal Medical and Chirurgical Society of London, to demonstrate and explain the theory that bilateral spasm and bilateral paralysis of the larynx may result from the pressure of an aneurism, or other tumor, upon the pneumogastric nerve of one side only. Reference was made to two cases of aneurism, one of the innominata, one of the aorta, which caused bilateral palsy of the larynx during life. After death, however, it was found that the vagus of one side only was involved. In both cases the laryngeal muscles of both sides were paralyzed, and Johnson's theory was that this was due on one side to direct pressure; on the other to reflex action. Dr. Johnson further said that pressure on one recurrent nerve (which is supplied with efferent motor fibres only, originating in the spinal accessory), will cause direct unilateral paralysis of the larynx, but cannot cause bilateral spasm or bilateral palsy. On the other hand, pressure upon the vagus involving its afferent fibres may cause both spasm and palsy of bilateral character. The reason for this bilateral action he considered to be the intimate connection between the nerve-nuclei of the two sides, thus according with the views of Dr. Broadbent. Dr. Lockhart Clarke has demonstrated that three sets of commissural fibres connect the nerve-nuclei of the origin of the spinal accessory, and this fact was also used by Dr. Johnson in support of his theory. At the same meeting, Dr. Powell stated that he had obtained like results in the dog, but called attention to the difference which exists in different animals, and added that faradization of one recurrent in the cat produced abduction of both cords. "Dr. John Reid," said Dr. Powell, "produced bilateral spasm in the larynx of the dog, by galvanizing one recurrent." Some years ago, Dr. Weir Mitchell discovered a remarkable chiasm in the laryngeal nerves of the turtle, and also experimented upon the cat and rabbit, but without result. He did not examine the dog. These statements of the English physicians, therefore, prompted his proposal that Dr. Keen should aid him in new experiments to determine these assertions in case of the dog. A few days later, Dr. Keen was invited to attend the execution of Heidenblut, and thereupon came the idea of making these experiments upon the human body directly after death.

Within thirty minutes after the execution of the criminal, Dr. Keen carefully dissected out the pneumogastric of one side, insulated the nerve by means of strips of pure rubber cloth, and then applied the two wires of the battery directly to the nerve. Meanwhile, Dr. Seiler attempted to watch the action of the larynx in the ordinary manner; but the hyoid bone of the subject had been fractured, thus closing the pharynx. Besides this, the throat of the dead man was occluded by a tenacious mucus, so that it was impossible to see the larynx. Dr. Keen therefore made an incision just above the thyroid cartilage, large enough only to admit the mirror, and recommenced the faradization of the nerves. I should remark that the vertebræ were neither fractured nor dislocated. This is the third case of judicial hanging in which Dr. Keen has verified this condition of the spinal column. The sterno-cleido-mastoid muscle of the right side had, however, been completely torn asunder by the pressure of the rope, the sheath being the only bond of union. Both vagi were apparently uninjured, and the recurrent nerves had been untouched by the rope. Upon re-applying the current, the vagus responded perfectly; but only one side of the larynx was affected. In spite of a forty cell current, and in spite of the afferent fibres which this nerve contains, no other result could be produced. Bilateral action of the vocal cords by galvanization of one recurrent nerve could take place only in case a chiasm existed between the laryngeal nerves (because this nerve contains only efferent motor fibres), and no such chiasm was found; only one nerve responded. Except it be that in this case the nerve centres were injured by the pressure of the rope, thus preventing a transmission of the current from one set of nerve-nuclei to the other, these results would seem to refute the assertions of Dr. Johnson.

Dr. Keen next galvanized the phrenic. The nerve did not respond. He then applied the current to the intercostal muscles, and proved most satisfactorily that the internal intercostals are muscles of inspiration and that the external intercostals are muscles of expiration; the former lifting the ribs, the latter depressing them. This manner of testing these muscles, which Dr. Keen was probably the first to attempt, will undoubtedly decide the question as to their real function, touching which there has been so much confusion of opinion. He also tested some of the facial muscles in relation to the part they take in expression, and found that the pyramidalis nasi, as stated by Darwin, is a direct antagonist to the occipito-frontalis.

Dr. Keen read a paper giving the results of his experiments upon Heidenblut, before the college of Physicians on Wednesday evening last. The details will therefore soon be published. Dr. Keen closed his paper by calling attention to the important results which this new method of investigation, namely, faradization of the muscles of the recently dead, promises to yield.

"In the living body, it is almost impossible to obtain the action of a single muscle, especially in the face, where the emotions, as pain, amusement, etc., involuntarily arouse the action of other muscles. In the recently dead, the results will be far more accurate, and therefore to the anatomist, physiologist, and artist, are of the greatest interest and importance."

UNGENANNT.

Philadelphia, Feb. 5, 1875.

